# Transcriptional expression of secondary resistance genes *ccdB* and *repA2* is enhanced in presence of cephalosporin and carbapenem in *Escherichia coli*

**DOI:** 10.1186/s12866-021-02136-y

**Published:** 2021-03-09

**Authors:** Somorita Baishya, Chandrayee Deshamukhya, Jayalaxmi Wangkheimayum, Bhaskar Jyoti Das, Anand Anbarasu, Anupam Das Talukdar, Amitabha Bhattacharjee, Manabendra Dutta Choudhury

**Affiliations:** 1 Department of Life Science and Bioinformatics, Assam University, Silchar, India; 2 Department of Microbiology, Assam University, Silchar, India; 3 Medical & Biological Computing Laboratory, School of Biosciences & Technology, VIT University, Vellore, India

**Keywords:** *repA2*, *ccdB*, Secondary resistance, Cephalosporin, Carabapenem, *Escherichia coli*

## Abstract

**Background:**

The issue of carbapenem resistance in *E.coli* is very concerning and it is speculated that cumulative effect of both primary resistance genes and secondary resistance genes that act as helper to the primary resistance genes are the reason behind their aggravation. Therefore, here we attempted to find the role of two secondary resistance genes (SRG) *ccdB* and *repA2* in carbapenem resistance in *E. coli* (CRE). In this context influential genes belonging to secondary resistome that act as helper to the primary resistance genes like *bla*_NDM_ and *bla*_CTX-M_ in aggravating β-lactam resistance were selected from an earlier reported in silico study. Transcriptional expression of the selected genes in clinical isolates of *E.coli* that were discretely harboring *bla*_NDM-1_, *bla*_NDM-4_, *bla*_NDM-5_, *bla*_NDM-7_ and *bla*_CTX-M-15_ with and without carbapenem and cephalosporin stress (2 μg/ml) was determined by real time PCR. Cured mutants sets that were lacking (i) primary resistance genes, (ii) secondary resistance genes and (iii) both primary and secondary resistance genes were prepared by SDS treatment. These sets were then subjected to antibiotic susceptibility testing by Kirby Bauer disc diffusion method.

**Results:**

Out of the 21 genes reported in the in silico study, 2 genes viz**.** *repA2* and *ccdB* were selected for transcriptional expression analysis. *repA2,* coding replication regulatory protein, was downregulated in response to carbapenems and cephalosporins. *ccdB*, coding for plasmid maintenance protein, was also downregulated in response to carbapenems except imipenem and cephalosporins. Following plasmid elimination assay increase in diameter of zone of inhibition under stress of both antibiotics was observed as compared to uncured control hinting at the reversion of antibiotic susceptibility by the-then resistant bacteria.

**Conclusion:**

SRGs *repA2* and *ccdB* help sustenance of *bla*_NDM_ and *bla*_CTX-M_ under carbapenem and cephalosporin stress.

**Supplementary Information:**

The online version contains supplementary material available at 10.1186/s12866-021-02136-y.

## Background

Morbidity and mortality rates due to Gram negative bacterial infections is increasing due to the global threat of antibiotic resistance [1]. This issue is a bane to health care industry as well as to economy [1, 2] and dearth in production of newer and efficient antibiotics is contributing to its escalation [3–5]. Dissemination of resistance as featured by Enterobacteriaceae to cephalosporins and carbapenems is concerning [6, 7] and incidentally, carbapenems happen to be the drugs of last resort for treating Gram negative bacterial infections [8–10].

The β- lactam ring of cephalosporins are hydrolyzed by CTX-M, the most prevalent and clinically relevant extended-spectrum β lactamases (ESBLs) while, carbapenems in carbapenem resistant Enterobacteriaceae (CRE) are inactivated by acquiring genes that code for carbapenemases [7, 11, 12]. Reports of dissemination of CTX-M from Europe to Asia indicates its wide spread occurrence and also reflects the associated apprehension [7, 13, 14]. Following the first report of *bla*_CTX-M-15_ from India many reports of its prevalence was reported from this sub-continent [14]. Likewise, among the commonly produced carbapenemases by CRE, *bla*_NDM_ (New Delhi metallo-beta-lactamase) is one of the most widespread variant in this part of the world [15, 16]. After the report of first incidence in 2008, several works reporting the mayhem of *bla*_NDM_ has surfaced [17–19]. The probable ineffectiveness of carbapenems is alarming [8, 18] and hence, the need of the hour is to identify newer antibiotic targets that could efficiently reduce the issue in concern [5].

At the onset of developing newer therapeutic regime it is imperative to understand the genetic and molecular mechanisms involved in a system [20]*.* Holistic models like gene networks designed using high throughput technologies can be used to identify genes, their molecular and biological functions that are essential in a system. Elimination of the same genes from the system will be helpful in confirming their role in the system, thereby posing them as potential novel antimicrobial targets [4, 21].

The report published on gene network analysis of CRE harboring *bla*_NDM_ unveiled that apart from the genes primarily associated with CRE like *bla*_NDM_, *ampC* some other genes, that are apparently non-essential in imparting carbapenem resistance, play influential role in the system [22]. These genes help the bacteria to survive under therapeutic stress of carbapenems and cephalosporins. Such genes are designated as “Secondary Resistance Genes” (SRG) [4]. This study is a first attempt to validate of the role of SRGs obtained by gene network analysis of CRE. Here the roles of a few selected SRGs (*repA2* and *ccdB*) were deciphered by studying their transcriptional pattern in response to carbapenems and cephalosporins stress and performing gene elimination assays. Since carbapenem and cephalosporin resistances are plasmid- mediated resistance therefore, *repA2* gene which is associated with replication regulation and *ccdB* which associated with plasmid maintenance protein were selected for the study. Transcriptional response of these genes to antibiotic stress and change in antibiotic susceptibility pattern following gene elimination assays indicated their role as helper to primary resistance genes in aggravating carbapenem resistance. The uniqueness of these genes in relation to homology with human proteins was also checked in order to establish these SRGs as newer antimicrobial targets that might be helpful in revoking carbapenem resistance.

## Results

### Transcriptional expression of *ccdB* and *repA2* genes with and without antibiotic stress

**Fig. 1:**
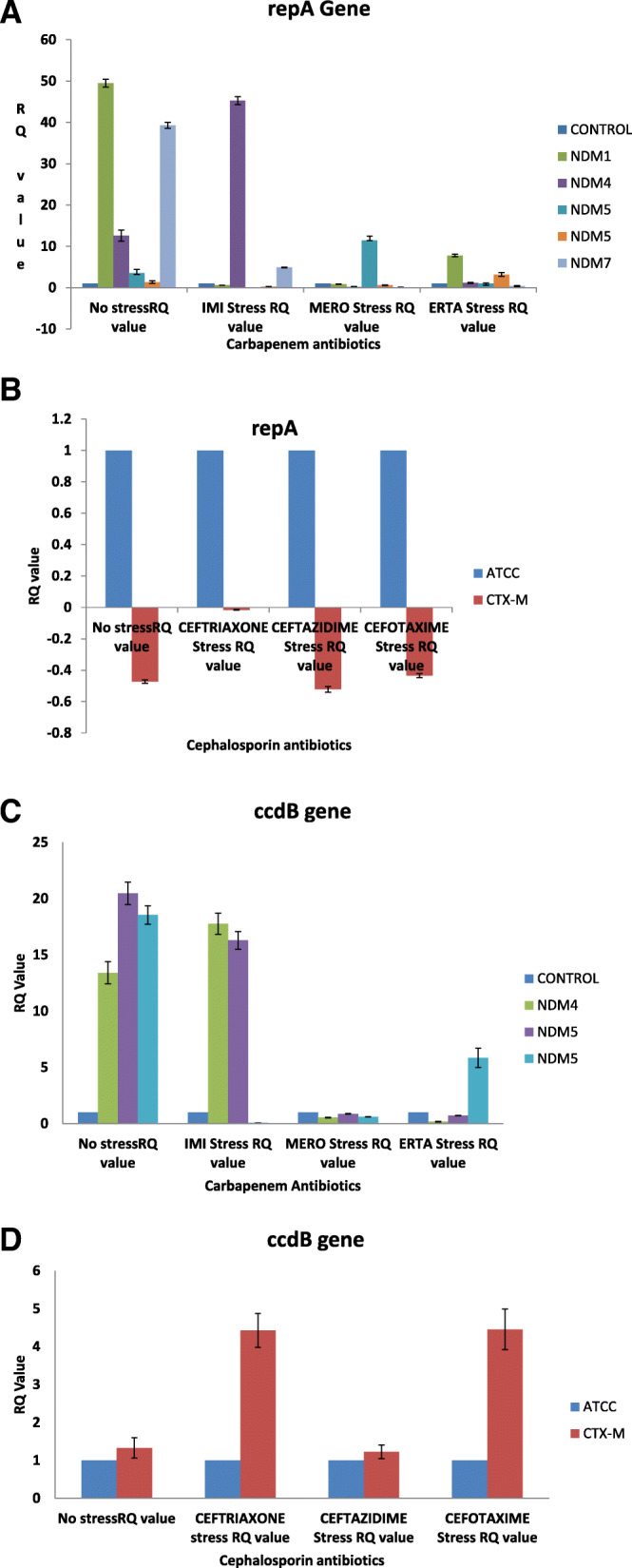
**a** Transcriptional response of *repA2* to carbapenems. **b** Transcriptional response of *repA2* to cephalosporins. c Transcriptional response of *ccdB* to carbapenems. d Transcriptional response of *ccdB* to cephalosporins

*ccdB* and *repA2* genes showed distinct pattern of response in these selected isolates when exposed to carbapenems and cephalosporins (Fig. 1a, b, c, d). However, the expressional pattern upon exposure to quinolones and aminoglycosides were not encouraging as no specific pattern of expression was observed (Supplementary material Fig. 1a, b). Upregulation of *repA2* gene was observed in *bla*_NDM-1_, *bla*_NDM-4_, *bla*_NDM-5_ and *bla*_NDM-7_ without antibiotics stress. Down regulation of *repA2* gene was observed in all the isolates on exposure to 2 μg/ml of meropenem. Stress of 2 μg/ml of ertapenem showed upregulation of *repA2* as compared to control except in isolates harboring *bla*_NDM-7_ however, downregulation of the same gene was seen in all the isolates when compared with the isolates without stress. Under ertapenem pressure the maximal expression value of *repA2* was observed in *bla*_NDM-1_ (RQ = 7.77) followed by *bla*_NDM-5_ (RQ = 3.18). In case of 2 μg/ml of imipenem stress, downregulation of *repA2* was observed in isolates carrying *bla*_NDM-1_ and *bla*_NDM-5_, while, isolates carrying *bla*_NDM-4_, *bla*_NDM-7_ showed upregulation. However, if compared to without stress isolates, *repA2* was downregulated in isolates carrying *bla*_NDM-7_. ANOVA test showed expression of *repA2* in response to carbapenems in *bla*_NDM_ isolates were statistically significant (p- value = 0.05). Downregulation of *repA2* was also seen in isolates that were harboring *bla*_CTX-M-15_ both when no cephalosporin stress was given as well as when 2 μg/ml of cephalosporins viz. ceftazidime, cefotaxime and ceftriaxone stress was applied. ANOVA test showed that *p*-value of *repA2* as 0.99 in response to cephalosporins in *bla*_CTX-M-15_ isolates. Upregulation of *ccdB* gene was observed in isolates that were bearing *bla*_NDM-4_, *bla*_NDM-5_ and *bla*_CTX-M-15_ on which no antibiotic stress was applied. Imipenem stress of 2 μg/ml upregulated the expression level of *ccdB* in all the isolates and the maximal transcription level was shown by *bla*_NDM-4_ (RQ = 17.67). Meropenem and ertapenem stress of equal volume showed downregulation of the gene in all the isolates. ANOVA test showed that *p*-value of *ccdB* as 0.09 in response to carbapenems in *bla*_NDM_ isolates. Ceftazidime, cefotaxime and ceftriaxone stresses at 2 μg/ml showed upregulation of *ccdB* however, if compared with without stress isolates *ccdB* was downregulated. ANOVA test showed that *p*-value of *ccdB* as 0.67 in response to cephalosporins in *bla*_CTX-M-15_ isolates.

### Susceptibility testing of mutants lacking primary and secondary resistance genes

Complete elimination of primary resistance genes viz. *bla*_NDM_ and *bla*_CTX-M-15_ after 3 passages and secondary resistance genes viz. *ccdB* and *repA2* after single passage was confirmed by PCR assay. Hence 6 sets of mutants as mentioned in methodology section were obtained.

Cured mutants were subjected to disc diffusion method and their susceptibility to various carbapenems and cephalosporins were compared against an uncured control. The zone diameter had increased in all the mutant plates compared to the control plate indicating that elimination of the genes had revoked resistance of the isolates to both the antibiotic groups. The measurements of zone of inhibitions of the mutants and uncured isolates against each antibiotic are given in Table 1a and b.

**Table 1:** (a) and (b): Measurements of zone of inhibitions of the mutants and uncured isolates against each antibiotic

(a)						
Sl. No	Antibiotic	Uncured isolate (mm)	Δ *bla*NDM		Δ *bla*NDM Δ*repA2* (mm)	Δ *bla*NDM Δ*ccdB* Δ*repA2* (mm)
1	Meropenem	0	12		12	21
2	Imipenem	16	19		19	25
3	Ertapenem	0	13		13	27
4	Ampicillin	0	0		0	0
5	Ceftazidime	0	0		0	25
6	Amikacin	21	30		30	26
7	Gentamicin	25	24		24	22
8	Ciprofloxacin	0	0		0	32
9	Cefepime	0	0		0	26.5
(b)						
Sl. No	Antibiotic	Uncured isolate (mm)	Δ *bla*CTX-M-15 (mm)	Δ *bla*CTX-M-15 Δ*ccdB* (mm)	Δ *bla*CTX-M-15 Δ*repA2* (mm)	Δ *bla*CTX-M-15 Δ*ccdB* Δ*repA2* (mm)
1	Ciprofloxacin	0	0	5	5	32
2	Gentamicin	22	23	29	30	24
3	Imipenem	18	20	23	21	25
4	Meropenem	18	20	23	20	26
5	Amikacin	20	26	30	30	28
6	Ertapenem	17	20	23	22	25
7	Ampicillin	0	5	8	8	16
8	Ceftazidime	10	13	18	18	25
9	Cefepime	0	8	13	13	26

## Discussion

The issues of antimicrobial resistance specifically towards carbapenems require immediate attention. Developing a holistic model to visualize and decipher the role of genes of secondary resistome along with the primary resistance genes is explicit [4, 22]. Genes associated with CRE is considered in this study. Reports of association of SRGs with carbapenem resistance are not found yet.

From the 21 genes that had been streamlined from in silico analysis [22] two genes viz. *ccdB* (cluster 19) involved with plasmid maintenance protein and *repA2* (cluster 33) associated with replication regulatory protein have been filtered out for in vitro transcriptional analysis. There are no reports of these genes of secondary resistome to be involved with carbapenem resistance owing to the presence of *bla*_NDM_ genes.

The response patterns of expression profiles of *repA2* and *ccdB* genes on carbapenem and cephalosporin exposure on clinical isolates of *E.coli* discretely harboring *bla*_NDM*-1*_*, bla*_NDM*-4*_*, bla*_NDM*-5*_*, bla*_NDM*-7*_ and *bla*_CTX-M-15_ were interesting. Elimination of these SRGs is essential for prediction of their role in a system [23].

*repA2* gene in the clinical samples was down regulated on exposure to imipenem, meropenem and ertapenem and under ertapenem pressure maximal expression was seen in the isolate harbouring *bla*_NDM*-1*_. Similarly, on various cephalosprins exposure *repA2* gene was down regulated. Eliminating *repA2* genes also showed changes in the susceptibility pattern against the antibiotics in concern. *repA2*, a replication initiation protein, controls replication of the plasmids belonging to IncFII group. The expression of this gene is regulated by the promoters situated at its upstream region. 5′ end of RNA-CX, a constitutively produced transcript, encodes *repA2* which acts as a repressor. This repressor regulates expression of RNA-A, another transcript. RNA-E, produced by the anti-parallel DNA strand regulates the translation of both RNA-CX and RNA-A. Hence, RNA-E directly interacts with both RNA-CX and RNA-A and can inhibit their translation. In this way *repA2* regulates plasmid copy number [24, 25]. Reports also show that mutations in *repA2* gene can increase plasmid copy number [26] while its disruption can stop the plasmid replication [27].

Congruent to literature, change in transcriptional response pattern of *repA2* upon antibiotic exposure as well as change in antibiotic susceptibility pattern upon its elimination was seen in this study. Due to these changes *repA2* can be considered as an indicator of antibiotic stress [28]. Since, plasmids play a crucial role in dissemination of antibiotic resistance and increase in the plasmid copy number gives bacteria better chances to adapt to antibiotic stress [29], therefore, disrupting *repA2* gene activity [27] might inactivate replication. The increase in diameters of zone of inhibition upon elimination of *repA2* as seen in this study indicates that elimination of this gene from *E.coli* system leads to loss of plasmid thereby rendering *E. coli* of clinical relevance non-pathogenic. These characteristics make *repA2* a good candidate for designing gene marker [30] From this finding it can be considered that *repA2* gene plays a crucial role as helper to the primary carbapenem resistance genes and its elimination might also be able to revoke carbapenem resistance and thus help in solving the problem of antibiotic resistance [31].

The *ccdB* gene, associated with plasmid maintenance, was down regulated when all the above mentioned clinical isolates were put under meropenem, ertapenem and all the three cephalosporins pressure. However, imipenem stress showed over expression of *ccdB* gene. Elimination of *ccdB* genes showed increase in the zone of inhibition indicating changes in the susceptibility pattern against the antibiotics in concern. The *ccdB* operon (control of cell death), a type of plasmid addiction system (PAS), is encoded by IncF plasmid to maintain plasmid stability in *E. coli* [32]. The operon consists of *ccdB*A and *ccdB* genes that codes for a toxin-antitoxin system which work in unison to maintain plasmid replication during cell division in host [33, 34]. The *ccdB* gene encodes DNA gyrase poison that can induce double strand breaks in *E. coli*, ultimately killing it [35]. This mechanism is activated only when the plasmid copy numbers decreases. Typically, gene *ccdB*A binds tightly to *ccdB* and encodes an antitoxin that inhibits the toxic activity of *ccdB* gene. On losing F-plasmid, Lon protease, a substrate for *ccdB*A, degrades it leaving *ccdB* free to act upon DNA gyrase [32, 35]. GyrA subunit is an antibiotic target for quinolones, however, quinolone resistant bacteria have no effect on *ccdB* indicating that *ccdB* and GyrA subunits interact at different sites [35]. Increase in the diameter of zone of inhibition upon elimination of *ccdB* genes as seen in this study hints that this gene supports the survival of *E. coli* under therapeutic stress condition and on its elimination the toxic function of this gene activates in order to maintain the PAS. All these findings make *ccdB* gene, a member of PAS, an interesting antibiotic target that could yield desirable results against carbapenem resistance [32, 36].

## Conclusion

As real is the issue of carbapenem resistance, so is the need to search and design newer antibiotic targets. Since the genes of secondary resistome act as helper to the genes primarily associated with carbapenem resistance, they can be regarded as potential drug targets for designing newer antibiotics. Revokement of resistance upon elimination of *ccdB* and *repA2* genes indicate that genes of secondary resistome do act as helper to primary resistance genes like *bla*_NDM_ thereby aggravating carbapenem resistance.

## Materials and methods

### Selection of genes from in silico analysis of gene network of carbapenem resistant enterobacteriaceae

From the in silico report published [22] in 2019 on potential drug targets against CRE using gene network analysis, *ccdB* and *repA2* from clusters 19 and 33 respectively were selected for transcriptional expression analysis with and without antibiotic stress. *ccdB* gene is associated with plasmid maintenance whereas *repA2* regulates plasmid replication.

### Bacterial isolates

Each clinical isolates of *E. coli* selected for the study were individually harboring *bla*_NDM-1_, *bla*_NDM-4_, *bla*_NDM-5_, *bla*_NDM-7_ and *bla*_CTX-M-15_. Single strains *bla*_NDM-1_, *bla*_NDM-4_, *bla*_NDM-7_ and *bla*_CTX-M-15,_ and two strains of *bla*_NDM-5_ were used for the study. *E. coli* ATCC 25922 was used as control for the study. PCR was done to confirm that all the isolates possessed *bla*_NDM_, *bla*_CTX-M_, *ccdB* and *repA2*genes. Primers were used in this study to confirm their presence is given in Table 2.

**Table 2:** Primers used in this study

Primer pairs	Target	Sequence	Reference
NDM-F NDM-R	*NDM*	5′-GGGCAGTCGCTTCCAACGGT-3′ 5′-GTAGTGCTCAGTGTCGGCAT-3’	[37]
CTX-M-F CTX-M-R	*CTX-M*	5’-CGCTTTGCGATGTGCAG-3′ 5′-ACCGCGATATCGTTGGT-3’	[37]
ccdB-F ccdB-R	*ccdB*	5’- CGAAGCGGGAATGCGGTAAT-3′ 5′-CATCCTGCTATCTGGCTCCT-3′	This study
repA2-F repA2-R	*repA2*	5′-AGGCCCGGTTAAAAGACAGG-3′ 5′-CAAAGTCGCTCTGCGTTTCA-3′	This study

### Transcriptional expression of *ccdB* and *repA2* genes with and without antibiotic stress

The transcriptional response of *ccdB* and *repA2* genes in the isolates was determined by quantitative real time PCR. The isolates harboring *bla*_NDM_ and *bla*_CTX-M-15_ were cultured overnight with 2 μg/ml carbapenem (imipenem, meropenem, ertapenem), cephalosporin (cefotaxime, ceftriaxone, ceftazidime), quinolone (cipfrofloxacin, norfloxacin) and aminoglycoside (gentamicin, amikacin) stress respectively. The isolates were also cultured under normal condition i.e. without any antibiotic pressure. Total mRNA was isolated from the overnight cultures grown to log phase using RNeasy kit (Qiagen, India). cDNA was prepared from the isolated mRNA using the Quanti Tect Reverse Transcription kit (Qiagen, India) following the manufacturer’s protocol. The cDNA generated was quantified by Picodrop (Pico 200, Cambridge, UK) and was then used as template for quantitative real time PCR using the Power Sybr Green Master Mix (Applied Biosystem, Warrington, UK) in Step One Plus realtime detection system. The relative quantity of the expression of *ccdB* and *repA2* genes in the isolates was evaluated using the ΔΔct method in reference to the corresponding expression of the genes in *E. coli* ATCC 25922. 30S ribosomal protein subunit *rpsl* gene of *E. coli* was also used in parallel as an internal control throughout the reactions.

### Susceptibility testing of mutants lacking primary and secondary resistance genes

The isolates harboring both the primary (*bla*_NDM_ and *bla*_CTX-M-15_) and secondary (*ccdB* and *repA2*) resistance genes were eliminated by treatment with SDS. Randomly five to six single colonies of each of the isolates was inoculated in fresh 5 ml Luria Bertani broth supplemented with SDS consecutively ranging from a concentration of 2 to 8% and incubated overnight. The following sets of cured mutants were generated:

i) *E. coli* Δbla_NDM_ ΔccdB
ii) *E. coli* Δ bla_NDM_ ΔccdB ΔrepA2
iii) *E. coli* Δbla_CTX-M-15_
iv) *E. coli* Δbla_CTX-M-15_ ΔccdB
v) *E. coli* Δbla_CTX-M-15_ ΔrepA2
vi) *E. coli* Δbla_CTX-M-15_ ΔccdB ΔrepA2

All the above sets of mutants were then tested for antibiotic susceptibility by Kirby Bauer disc diffusion method. The antibiotics used were imipenem, meropenem, ertapenem, cefotaxime, cefepime, ceftazidime, ciprofloxacin, ampicillin, gentamicin and amikacin respectively. The diameter of the zone of inhibition of the mutants against each antibiotic was measured and compared to the diameter of zone of inhibition of the uncured isolate.

### Statistical analysis

The changes in expression of *bla*_NDM_ and *bla*_CTX-M-15_ in response to different carbapenem and cephalosporin stresses at 2 μg/ml concentration were analyzed using one-way ANOVA. Differences were considered statistically significant at when *p* value ≤0.05.

## Supplementary Information

**Additional file 1: Supplementary material Fig A.** Transcriptional response of *repA2* to aminoglycosides and quinolones. **Fig B.** Transcriptional response of *ccdB* to aminoglycosides and quinolones

## Data Availability

The datasets used and analysed in the current study are available from the corresponding author upon request.

## Abbreviations

CRE: Carbapenem resistant Enterobacteriaceae
ESBL: Extended-spectrum β lactamases
PAS: Plasmid addiction system
PCR: Polymerase chain reaction
RNA: Ribonucleic acid
SDS: Sodium dodecyl sulfate
SRG: Secondary resistance genes
