# Supplementary material for: Transcriptional expression of secondary resistance genes ccdB and repA2 is enhanced in presence of cephalosporin and carbapenem in Escherichia coli
Source: BMC Microbiol. 2021 Mar 9;21:79. doi: 10.1186/s12866-021-02136-y (PMC7941987; doi:10.1186/s12866-021-02136-y)
Supplement: Supplementary file 1 — Additional file 1: Supplementary material Fig A. Transcriptional response of repA2 to aminoglycosides and quinolones. Fig B. Transcriptional response of ccdB to aminoglycosides and quinolones [file 12866_2021_2136_MOESM1_ESM.docx]

Supplementary material

Fig A: Transcriptional response of *repA2* to aminoglycosides and quinolones

Fig B: Transcriptional response of *ccdB* to aminoglycosides and quinolones
